# Glucocorticoid-Induced Muscle Satellite Cell-Derived Extracellular Vesicles Mediate Skeletal Muscle Atrophy via the miR-335-5p/MAPK11/iNOS Pathway

**DOI:** 10.3390/biom15081072

**Published:** 2025-07-24

**Authors:** Pei Ma, Jiarui Wu, Ruiyuan Zhou, Linli Xue, Xiaomao Luo, Yi Yan, Jiayin Lu, Yanjun Dong, Jianjun Geng, Haidong Wang

**Affiliations:** 1 College of Veterinary Medicine, Shanxi Agricultural University, Jinzhong 030801, China; mapei@sxau.edu.cn (P.M.); t20225022@stu.sxau.edu.cn (J.W.); s20222405@stu.sxau.edu.cn (R.Z.); xuelinli@sxau.edu.cn (L.X.); xmluo@sxau.edu.cn (X.L.); yanyi@sxau.edu.cn (Y.Y.); jiayinlu@sxau.edu.cn (J.L.); gengjianjun@sxau.edu.cn (J.G.); 2 College of Veterinary Medicine, China Agricultural University, Beijing 100193, China; yanjund@cau.edu.cn

**Keywords:** extracellular vesicles, glucocorticoids, inducible nitric oxide synthase, miRNA, muscle satellite cells

## Abstract

Prolonged high-dose administration of synthetic glucocorticoids (GCs) leads to limb muscle atrophy and weakness, yet its underlying mechanisms remain incompletely understood. Muscle fibers and muscle satellite cells (MSCs) are essential for skeletal muscle development and associated pathologies. This study demonstrates that dexamethasone (Dex) induced MSC-derived extracellular vesicles (EVs) impair myogenesis in muscle fiber-like cells (MFLCs) via inducible nitric oxide synthase (iNOS) suppression. High-throughput sequencing revealed a marked upregulation of miR-335-5p in MSC-derived EVs following Dex treatment. Mechanistically, EV miR-335-5p targeted MAPK11, leading to iNOS downregulation and subsequent UPS activation in MFLCs, which directly promoted muscle protein degradation. Collectively, our findings identify the EV miR-335-5p/MAPK11/iNOS axis as a critical mediator of GC-induced muscle atrophy, offering novel insights into therapeutic strategies targeting EV-mediated signaling in muscle wasting disorders.

## 1. Introduction

Synthetic glucocorticoids (GCs), such as dexamethasone (Dex), are among the most commonly prescribed drugs for treating various inflammatory and autoimmune diseases [1]. Since the discovery of *coronavirus disease 2019* (*COVID-19*) in 2020, GCs have been widely used worldwide as an effective treatment for respiratory complications caused by *COVID-19* [2]. However, high-dose and long-term GC administration can lead to multiple adverse effects, particularly muscle atrophy [3]. GCs exert potent anti-inflammatory effects by inhibiting inducible nitric oxide synthase (iNOS), but this mechanism can also lead to adverse effects such as GC-induced myopathy [4,5]. However, GCs also exhibit catabolic effects by activating proteolytic pathways, including the ubiquitin–proteasome system, leading to enhanced muscle protein degradation [6]. Specifically, GCs upregulate muscle-specific E3 ubiquitin ligases, such as *FBXO32* (*MAFbx*/*Atrogin-1*) and *TRIM63* (*MuRF1*), thereby promoting protein degradation [7]. Concurrently, GCs stimulate Smad2/3 phosphorylation and Akt dephosphorylation, which amplifies ubiquitin–proteasome activity and ultimately accelerates muscle atrophy progression [8]. These processes are typically regulated by specific transcription factors, notably forkhead box O (FoxO) family members. Under normal conditions, FoxO proteins are phosphorylated and inactivated by Akt in the cytoplasm. However, GCs promote FoxO activation, which subsequently modulates the expression of target genes involved in skeletal muscle pathology [8].

Skeletal muscle possesses a precisely organized arrangement of muscle fibers (myofibers or muscle cells) and connective tissue, with overall muscle size determined by the number and size of individual muscle fibers [9]. Muscle satellite cells (MSCs) are quiescent myogenic progenitor cells located between the sarcolemma and basal lamina of muscle fibers, possessing proliferative and differentiation potential [10]. MSCs secrete a substantial number of exosomes, a specialized subtype of extracellular vesicles (EVs) with diameters ranging from 30 to 150 nm [11,12]. Exosomes are bilayer lipid membrane-enclosed vesicles that carry various nucleic acids, including DNA (both single-stranded and double-stranded DNA) and RNA (such as lncRNA, miRNA, mRNA, and rRNA), among which miRNA (microRNA) represents a key functional component [13]. The lipid bilayer membrane of exosomes effectively shields miRNA from RNase degradation, ensuring its stable delivery to recipient cells and subsequent regulation of gene expression [14]. Notably, the composition of exosomes can dynamically change in response to external stimuli or physiological conditions, thereby modulating their functional roles in intercellular communication [15]. Emerging evidence indicates that MSC-derived exosomes possess therapeutic potential for mitigating muscle atrophy and fibrosis [16]. In idiopathic inflammatory myopathies, plasma-derived exosomes carrying miR-335-5p are selectively upregulated in dermatomyositis, suggesting that exosomes may serve as potential biomarkers in muscle diseases [17]. Numerous studies have demonstrated that GCs impair MSC function, leading to skeletal muscle atrophy [18,19]. The potential role of GCs in mediating skeletal muscle atrophy through the impairment of MSC function and subsequent alteration of miRNA profiles in MSC-derived EVs remains to be elucidated.

This study reveals the molecular mechanism by which Dex-induced MSC-derived EVs regulate myogenesis in MFLCs through the miR-335-5p/MAPK11/iNOS signaling pathway, providing a theoretical basis for elucidating the pathogenesis of GC-induced muscle atrophy and developing novel therapeutic strategies.

## 2. Materials and Methods

### 2.1. Animals

Male C57BL/6 mice (SPF grade) aged 4–6 weeks were purchased from SPF Biotechnology Co., Ltd. (Beijing, China). All animal experiments were conducted following protocols approved by the Animal Care Committee of Shanxi Agricultural University (SXAU-EAW-2022M. QS. 006007247). The animals were maintained under pathogen-free conditions in temperature-controlled rooms with a 12 h light/12 h dark cycle and provided with unrestricted access to food and water. Following a one-week acclimation period, the mice were utilized for further experiments.

### 2.2. Isolation, Culture, and Treatment of Primary Skeletal Muscle Cells

The isolation and improvement of MFLCs and MSCs were performed according to previously described methods [20]. Briefly, after anesthesia, the 4–6-week-old mice were euthanized via cervical dislocation, and the hindlimb skeletal muscle tissues were dissected and thoroughly minced (*n* = 10). The samples were pooled (*n* = 10) to ensure a sufficient cell yield-pre-experimental validation showed that ≥1 × 10^8^ cells (typically obtained at passage 3, P3) yielded approximately 1 × 10^9^ EVs particles/mL, enabling exosome isolation and miRNA sequencing while maintaining cellular homogeneity and avoiding passage-dependent alterations in EV cargo. The tissue was digested with Collagenase II (500 U/mL, Sigma-Aldrich, St. Louis, MO, USA) at 37 °C for 30 min. Digestion was terminated, and the supernatant was collected. A second digestion step was performed using a mixture of Collagenase D (1.5 U/mL, Roche Applied Science, Indianapolis, IN, USA) and Dispase II (2.4 U/mL, Sigma-Aldrich, St. Louis, MO, USA) at 37 °C for 60 min. Digestion was terminated, and the supernatant was collected. The cell suspension was resuspended in DMEM and sequentially filtered through 100 µm and 40 µm cell strainers. The filtered cells were centrifuged at 1000 rpm for 5 min, and the pellet was collected. The cells were plated in culture dishes and left undisturbed for two 30 min intervals. After each interval, the supernatant was collected and centrifuged (1000 rpm, 5 min), and the resulting pellet was plated in new dishes to obtain MSCs. Adherent cells were gently detached and centrifuged (1000 rpm, 5 min) to collect MFLCs that were infinitely close to muscle fibers. MSCs and MFLCs were seeded on tissue culture plates coated with rat-tail collagen and cultured in growth medium (GM) containing high-glucose Dulbecco’s Modified Eagle Medium (DMEM), 20% fetal bovine serum (FBS), and 10% horse serum (HS). The cells were used between P2 and P3 (post-purification) after confirming that they were free of mycoplasma contamination.

MSCs (2 × 10^6^) were plated in 100 mm culture dishes, serum-starved in DMEM for 12 h, and then treated with 50 μM Dex (Beijing Solarbio Science & Technology Co., Ltd., Beijing, China) for 24 h [21]. The conditioned medium was harvested for EV isolation. MFLCs (2 × 10^5^ cells per well) were seeded in 6-well plates and serum-starved in DMEM for 12 h. The cells were then incubated with MSC-derived EVs (1 × 10^6^ particles/mL) for 24 h before collection for analysis or morphological evaluation.

### 2.3. Quantitative Real-Time Polymerase Chain Reaction (qRT-PCR)

Total RNA was extracted from the gastrocnemius muscle or cells using RNAiso Plus (Takara Bio Inc., Shiga, Japan) following the manufacturer’s instructions. For miRNA detection, RNA was reverse transcribed into cDNA using an miRNA 1st Strand cDNA Synthesis Kit (by stem–loop) (Nanjing Vazyme Biotech Co., Ltd., Nanjing, China). Quantitative PCR (qPCR) was performed using Antibody Dye-Based qPCR Premix (Monad Biotech Co., Ltd., Shanghai, China) and the ABI QS5 system to quantify the RNA levels of six selected miRNAs (Table 1). Expression levels were normalized to U6. For mRNA quantification, RNA was reverse transcribed into cDNA using HiScript III RT SuperMix for qPCR (Nanjing Vazyme Biotech Co., Ltd., Nanjing, China). qRT-PCR was performed with Antibody Dye-Based qPCR Premix (Monad Biotech Co., Ltd., Shanghai, China) and the Applied Biosystems QuantStudio 5 Real-Time PCR System (Thermo Fisher Scientific, Waltham, MA, USA). Primers were designed using online software (Primer3 Plus, v2.6.1, https://www.primer3plus.com/) and are listed in Table 1. Expression levels were normalized to β-actin. All reactions were performed in triplicate, and data were analyzed using the threshold cycle (ΔΔCT) method.

### 2.4. Western Blot

Protein samples were extracted from the cells using RIPA lysis buffer (Beyotime Biotechnology Co., Ltd., Shanghai, China) supplemented with a protease inhibitor (PMSF, Beijing Solarbio Science & Technology Co., Ltd., Beijing, China). Protein concentrations were determined using a BCA Protein Assay Kit (Beyotime Biotechnology Co., Ltd., Shanghai, China). A total of 30 μg of protein per sample was separated using SDS-PAGE and transferred onto PVDF membranes (MilliporeSigma, Merck KGaA, Darmstadt, Germany). The membranes were blocked with 5% non-fat milk at room temperature for 1 h, followed by incubation with primary antibodies and subsequently with HRP-conjugated secondary antibodies. The following primary antibodies were used: Anti-FBXO32 (A3193, 1:1000, Abclonal Technology Co., Ltd., Wuhan, China), Anti-TRIM63 (55456-1-AP, 1:1000, Proteintech Group, Inc., Wuhan, China), Anti-MYOG (sc-12732, 1:1000, Santa Cruz Biotechnology, Inc., Dallas, TX, USA), Anti-iNOS (TA0199S, 1:1000, Abmart, Inc., Shanghai, China), Anti-MAPK11 (17376-1-AP, 1:200, Proteintech Group, Inc., Wuhan, China), An-ti-TSG101 (28283-1-AP, 1:1000, Proteintech Group, Inc., Wuhan, China), Anti-Alix (12422-1-AP, 1:1000, Proteintech Group, Inc., Wuhan, China), Anti-CD63 (sc-5275, 1:500, Santa Cruz Biotechnology, Inc., Dallas, TX, USA), and Anti-GAPDH (10494-1-AP, 1:25000, Proteintech Group, Inc., Wuhan, China). Protein detection was performed using an ECL chemiluminescence kit (Abbkine Scientific Co., Ltd., Wuhan, China), and chemiluminescence intensity was analyzed using the Bio-Rad imaging system 6.0.0. Build 25.

### 2.5. Immunofluorescence

MFLCs and MSCs were fixed with 4% paraformaldehyde for 30 min and permeabilized with 0.5% Triton X-100 in PBS for 15 min. The cells were then blocked with 5% BSA in TBST at room temperature for 1 h. The cells were incubated overnight at 4 °C with the following primary antibodies diluted in 5% BSA: Anti-α-SMA (T55295, 1:200, Abmart, Inc., Shanghai, China), Anti-Vimentin (10366-1-AP, 1:200, Proteintech Group, Inc., Wuhan, China), Anti-PAX7 (ab187339, 1:200, Abcam, Cambridge, UK), and Anti-MYOD (TA7733M, 1:200, Abmart, Inc., Shanghai, China). The primary antibodies were detected using secondary antibodies conjugated with Alexa Fluor 568 or Alexa Fluor 488 (ab150077, ab150116, 1:400, Abcam, Cambridge, UK). Slides were mounted with anti-fade reagent containing DAPI (Beijing Solarbio Science & Technology Co., Ltd., Beijing, China) and imaged using a laser confocal microscope (OLYMPUS, FV1000, Tokyo, Japan).

### 2.6. EV Isolation and Characterization

EVs were obtained from the cell supernatant of MSCs (passages 2–3) cultured in DMEM for 2 days. Briefly, the supernatant was centrifuged at 300× *g* for 10 min to remove floating cells, followed by centrifugation at 2000× *g* for 10 min to eliminate cell debris. The supernatant was then centrifuged at 10,000× *g* for 30 min to remove apoptotic bodies and particles larger than 1 μm. The EVs were precipitated via ultracentrifugation at 100,000× *g* for 90 min (Beckman Coulter, Brea, CA, USA). The pellet was washed with PBS and ultracentrifuged again at 100,000× *g* to obtain EVs, which were then concentrated using an ultrafiltration tube (100 kDa MWCO, Catalogue No. UFC9100, MilliporeSigma, Merck KGaA, Darmstadt, Germany). The final EV pellet was resuspended in 100 μL of PBS and stored at −80 °C. Exosomes were observed using transmission electron microscopy (JEM-1200EX, Japan Electron Optics Laboratory, Tokyo, Japan) and quantified using nanoparticle tracking analysis (S/N 252, ZetaView, Inning am Ammersee, Germany). The EV protein markers TSG101, ALIX, and CD63 were identified using Western blot analysis.

### 2.7. miRNA Sequencing of MSC-Derived EVs

Sequencing analysis was performed at Lianchuan Biotechnology Co., Ltd. (Hangzhou, China). An miRNA sequencing library was generated using the standard Illumina (HiSeq 2500) workflow, including library preparation and sequencing. Libraries were constructed with TruSeq Small RNA Sample Prep Kits for Illumina^®^ (Illumina, San Diego, CA, USA) and sequenced on an Illumina HiSeq 2500 platform. Quality control metrics indicated that the MSC (EVs) sample generated 12.7 million raw reads with Q20 and Q30 scores of 96.48% and 94.78%, respectively, while the MSC-Dex (EVs) sample yielded 14.1 million raw reads with Q20 and Q30 scores of 96.64% and 95.19%, respectively. These results confirmed data integrity suitable for downstream analysis. Data analysis was performed using ACGT101-miR (v4.2). This included removing 3′ adapters and low-quality sequences to obtain clean data, retaining sequences 18–26 nt in length. miRNAs with read counts < 5 in all samples were excluded. The remaining sequences were aligned to mRNA, RFam, and Repbase databases (excluding miRNA) and filtered accordingly. Reads passing length and database filters were used for miRNA identification via alignment with precursors and the genome. The target genes of significantly differentially expressed miRNAs were predicted using TargetScan (v5.0) and miRanda (v3.3a). Differential expression analysis of miRNAs was performed with a significance threshold of |log2FC| > 1 (equivalent to a 2-fold change) and FDR (false discovery rate)-adjusted *p*-value < 0.05, using the Benjamini–Hochberg procedure to account for multiple comparisons.

### 2.8. EV Uptake

EVs were labeled with the green fluorescent dye PKH67 (MINI67, Sigma-Aldrich, St. Louis, MO, USA) for tracking purposes. EVs from 10 million cells were prepared according to the manufacturer’s instructions. PKH67-labeled EVs were added to MFLCs in 24-well plates at a concentration of 500 μL per well and incubated at 37 °C for 24 h. EV uptake was evaluated by imaging using a laser scanning confocal microscope (FV1000, Olympus, Tokyo, Japan).

### 2.9. miRNA Intervention

miR-335-5p mimics, inhibitors, and negative controls were synthesized by Hanbio Biotechnology Co., Ltd. (Shanghai, China). The transfection of miR-335-5p mimics and inhibitors was performed using Lipofectamine™2000 CD (Thermo Fisher Scientific, Waltham, MA, USA) following the manufacturer’s instructions.

### 2.10. Fluorescence In Situ Hybridization (FISH)

Cells on coverslips were fixed in 4% paraformaldehyde for 10 min and washed with PBS. The coverslips were incubated in 3% citric acid-diluted pepsin solution for 20 min, followed by three washes with distilled water. Pre-hybridization solution was applied to the coverslips for 4 h. Subsequently, the coverslips were incubated in hybridization solution at 42 °C overnight in the dark. The next day, the coverslips were washed four times (10 min each) with gradient saline–sodium citrate (SSC) buffer. Fluorescence images were acquired with a confocal laser microscope (FV1000, Olympus, Tokyo, Japan), followed by quantitative analysis of fluorescence intensity using ImageJ (v1.53a, NIH, USA).

### 2.11. Dual-Luciferase Reporter Assay

To identify the shared binding site between miR-335-5p and MAPK11, a luciferase reporter assay was conducted by Hanbio Biotechnology Co., Ltd. (Shanghai, China). MFLCs were transfected with a luciferase construct containing MAPK11, which harbored either the wild-type or mutated binding site. The cells were co-transfected with miR-335-5p or a negative control. After 6 h, the medium was replaced, and luciferase activity was measured 48 h post-transfection using a dual-luciferase reporter assay kit (Yeasen Biotechnology Co., Ltd., Shanghai, China).

### 2.12. Data Analysis

All experimental data are presented as the mean ± standard error of the mean (SEM), with each experiment independently repeated at least three times. In relative gene expression analysis, the mean value of the control group was normalized to 100% (or 1) as the baseline. Statistical analyses were performed using ImageJ 9.0 software (GraphPad Prism version 9, La Jolla, CA, USA). The Shapiro–Wilk test was used to assess normality, and Levene’s test was applied to evaluate the homogeneity of variance. For data meeting both the normality and homogeneity of variance assumptions, group comparisons were conducted using unpaired Student’s *t*-tests. Differences were considered significant at *p* < 0.05, denoted as * *p* < 0.05 and ** *p* < 0.01.

## 3. Results

### 3.1. Isolation and Characterization of Primary MFLCs and MSCs from Skeletal Muscle

**Figure 1:**
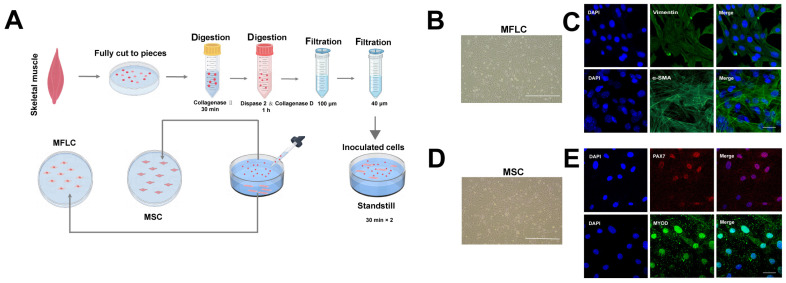
Characterization of Muscle fiber-like cells (MFLCs) and Muscle satellite cells (MSCs): (**A**) A schematic diagram of the MFLC and MSC primary isolation from mice skeletal muscle. (**B**) Representative images of MFLC morphology. Bar: 200 μm. (**C**) Immunofluorescence representative images of MFLC-specific markers (Vimentin/α-SMA). Bar: 50 μm. (**D**) Representative images of MSC morphology. Bar: 200 μm. (**E**) Immunofluorescence representative images of MSC-specific markers (PAX7 and MYOD). Bar: 50 μm.

To explore the mechanisms of glucocorticoid-induced skeletal muscle atrophy and better simulate the interaction between muscle fibers and muscle satellite cells in skeletal muscle, in this study, differential adhesion plating was employed to isolate different muscle cell populations from the primary culture of mouse skeletal muscle (Figure 1A). Under inverted microscopy, both MFLCs and MSCs exhibited spindle-shaped or fusiform morphologies (Figure 1B,D). Based on their adhesion properties, the first batch of rapidly adhering cells primarily consisted of fibroblast-like cells and myoblasts, (herein collectively referred to as muscle fiber-like cells (MFLCs)), while the slower-adhering cells were characterized as muscle satellite cells (MSCs) [20]. Previous studies (Gharaibeh et al.) have shown that primary MFLCs remain quiescent for the first 1–3 days after seeding before entering the cell cycle and undergoing 3–5 rounds of proliferation. Notably, even in growth medium, over 80% of MFLCs can fuse to form myotubes [20]. Immunostaining confirmed the successful isolation of MFLCs and MSCs. Primary MFLCs showed a high expression of the MFLC-specific markers Vimentin and α-SMA (Figure 1C). In contrast, MSCs exhibited a high expression of the MSC-specific markers PAX7 and MYOD (Figure 1E). These results collectively demonstrate the successful isolation and characterization of primary MFLCs and MSCs.

### 3.2. Characterization of Dex-Induced MSC-Derived EVs

**Figure 2:**
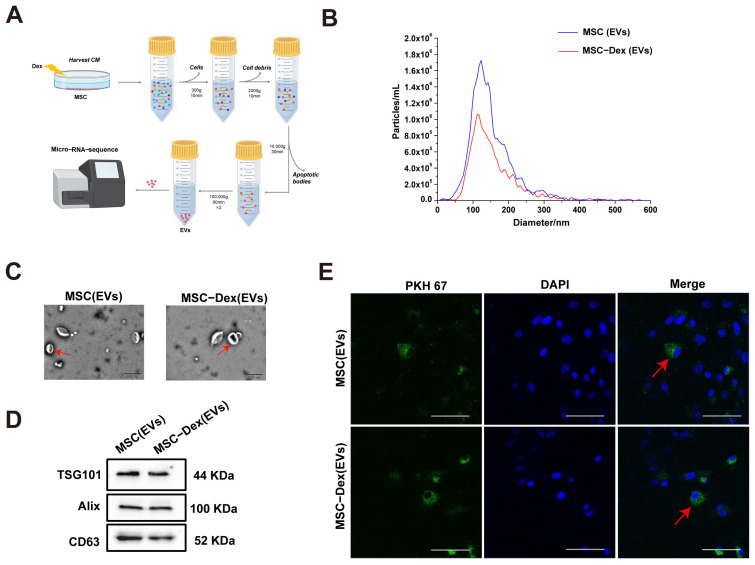
Identification and characterization of Extracellular Vesicle (EV) markers from MSC-derived EVs: (**A**) Schematic diagram of collecting MSC-derived EVs. (**B**) NanoSight detected the particle size and concentration of MSC or MSC-Dex-derived EVs. (**C**) Transmission electron microscopy (TEM) detected the morphology of each group. The red arrows indicate EVs. Bar: 100 nm. (**D**) Western blot analysis of EVs positive-specific markers (TSG101, Alix, and CD63) in EVs derived from MSCs of each group. Original Western blot images can be found in Figure S2. (**E**) Representative fluorescence microscope images of MFLCs with PKH67 (green)-labeled MSC or MSC-Dex-derived EVs. Red arrows indicate EV uptake. Bar: 200 μm.

MSCs possess proliferative and differentiation potential, and their EVs mediate intercellular communication. Therefore, MSC-derived EVs were isolated for further investigation (Figure 2A). The results demonstrated that the average vesicle diameters were 123.6 nm for untreated MSCs and 114.5 nm for Dex-treated MSCs. Moreover, the EV concentration in Dex-treated MSCs was significantly lower than that in untreated MSCs (Figure 2B). The vesicles exhibited a characteristic double-layer membrane spherical structure (Figure 2C), and the EV markers TSG101, Alix, and CD63 were positively expressed (Figure 2D). Additionally, PKH67-labeled EVs were internalized by MFLCs (Figure 2E). These data confirm the successful isolation of MSC-derived EVs and their uptake by MFLCs, providing a foundation for subsequent experiments.

### 3.3. Dex-Induced MSC-Derived EVs Cause Protein Degradation in MFLC via iNOS

**Figure 3:**
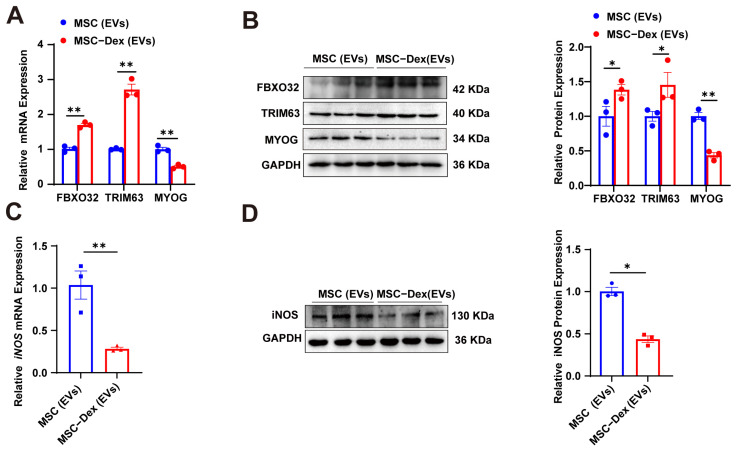
Dex-induced MSC-derived EVs cause protein degradation in MFLCs via iNOS: (**A**) qRT-PCR analysis of *FBXO32*, *TRIM63*, *MYOG*, and *iNOS* mRNA levels in MFLCs after 24 h of intervention with MSC or MSC-Dex-derived EVs. (**B**) Western blot analysis of FBXO32, TRIM63, and MYOG protein levels in MFLCs after 24 h of intervention with MSC or MSC-Dex-derived EVs. Original Western blot images can be found in Figure S3. (**C**) qRT-PCR analysis of *iNOS* mRNA expression in MFLCs after 24 h of intervention with MSC or MSC-Dex-derived EVs. (**D**) Western blot analysis of iNOS protein expression in MFLCs after 24 h of intervention with MSC or MSC-Dex-derived EVs. *n* = 3. The results are expressed as the mean ± SEM. * *p* < 0.05, ** *p* < 0.01, values significantly different from the corresponding control based on an unpaired *t*-test. Original Western blot images can be found in Figure S3.

To explore whether Dex-induced MSC-derived EVs could promote protein degradation in MFLCs, thereby impairing muscle growth, we isolated EVs from both untreated and Dex-treated MSCs and treated MFLCs with them. The results demonstrated that the EVs derived from Dex-treated MSCs significantly upregulated the expression of the atrophy-related genes *FBXO32* (Atrogin-1) and *TRIM63* (MuRF1) in MFLCs, while downregulating MYOG (myogenin) expression (Figure 3A,B). Furthermore, iNOS levels were also reduced following treatment with Dex-induced MSC-derived EVs (Figure 3C,D). It is well established that GCs exert their anti-inflammatory effects by suppressing iNOS expression, which also constitutes a key mechanism underlying GC-induced myopathy [4,5]. Here, we observed that Dex-induced MSC-derived EVs similarly downregulated iNOS, concomitant with enhanced proteolysis. These findings suggest that Dex-induced MSC-derived EVs may enhance protein degradation in MFLCs, potentially through an iNOS-mediated mechanism.

### 3.4. Dex Alters miRNA Cargo in MSC-Derived EVs

**Figure 4:**
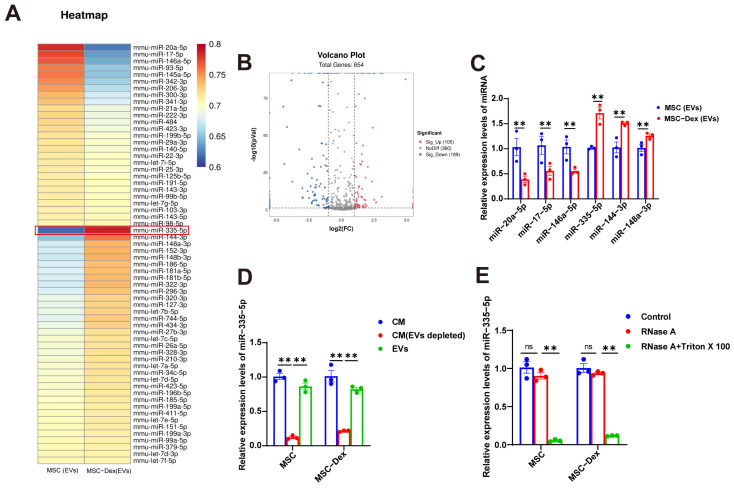
Dex alters miRNA cargo in MSC-derived EVs: (**A**) A differential miRNA clustering heatmap of MSC and MSC-Dex-derived EVs. (**B**) A differential miRNA volcano map of MSC or MSC-Dex-derived EVs. (**C**) qRT-PCR analysis of the first six miRNAs in MSC and MSC-Dex-derived EVs. (**D**) qRT-PCR analysis of miR-335-5p levels in the CM of MSC and depleted of EVs or by ultracentrifugation. (**E**) qRT-PCR analysis of miR-335-5p levels in MSC and MSC-Dex treated with control medium or RNase A (2 mg/mL) alone or in combination with Triton X-100 (0.1%) for 0.5 h. *n* = 3. The results are expressed as mean ± SEM. ** *p* < 0.01, ns, not significant, values significantly differ from those of the corresponding control, determined using an unpaired *t*-test.

It is well known that miRNAs are key molecules transferred by EVs to recipient cells. A miRNA-seq analysis was performed to identify differentially expressed miRNAs in EVs from untreated and Dex-treated MSCs. Heatmaps displayed all significantly differentially expressed miRNAs, and, miR-335-5p, the most significantly altered miRNA, was selected based on its ability to bind to target mRNA and suppress its expression (Figure 4A). Volcano plots identified 654 miRNAs in EVs from normal and Dex-treated MSCs, including 105 upregulated and 189 downregulated miRNAs, with 360 miRNAs showing no change (Figure 4B). The validation of six differentially expressed miRNAs (Figure 4C) revealed results consistent with the miRNA-seq data: miR-20-5p, miR-17-5p, and miR-146a-5p were reduced in EVs from Dex-treated MSCs compared to in normal MSC-derived EVs, whereas miR-335-5p, miR-144-3p, and miR-148a-3p were increased.

This confirmed the reliability of the miRNA sequencing data. We next measured the miR-335-5p levels in the total conditioned medium (CM), EV-depleted CM (ultracentrifugation), and EVs. miR-335-5p expression was significantly higher in the total CM and EVs than in the EV-depleted CM (Figure 4D). Notably, the miR-335-5p levels in the MSC-derived CM remained unchanged after RNase A treatment but were significantly reduced after RNase A and Triton X-100 treatment (Figure 4E). This indicates that extracellular miR-335-5p is encapsulated within membranes rather than being directly secreted, suggesting that miR-335-5p is primarily packaged in MSC-derived EVs. These findings demonstrate that Dex-induced MSC-derived EVs regulate iNOS through miR-335-5p, causing protein degradation in MFLCs.

### 3.5. miR-335-5p Targets MAPK11/iNOS, Inducing Protein Degradation in MFLCs

**Figure 5:**
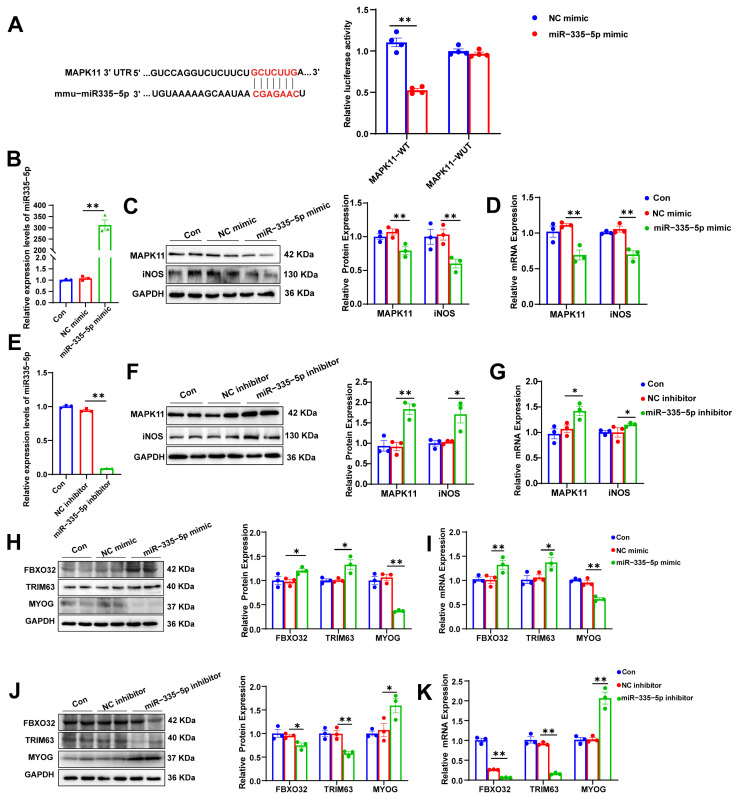
miR-335-5p targets MAPK11/iNOS, inducing protein degradation in MFLCs: (**A**) Structure and luciferase result of dual-luciferase reporter gene of the WT or MUT 3′ UTR of mouse *MAPK11*. (**B**) qRT-PCR analysis of miR-335-5p levels transfected with an miR-335-5p or NC mimic in MFLCs. (**C**) Western blot analysis of MAPK11 and iNOS protein levels in MFLCs of each group. Original Western blot images can be found in Figure S4. (**D**) qRT-PCR analysis of *MAPK11* and *iNOS* mRNA levels in MFLCs of each group. (**E**) qRT-PCR analysis of miR-335-5p levels transfected with an miR-335-5p or NC inhibitor in MFLCs. (**F**) Western blot analysis of MAPK11 and iNOS protein levels in MFLCs of each group. Original Western blot images can be found in Figure S4. (**G**) qRT-PCR analysis of *MAPK11* and *iNOS* mRNA levels in MFLCs of each group. (**H**) Western blot analysis of FBXO32, TRIM63, and MYOG protein levels transfected with an miR-335-5p or NC mimic in MFLCs. Original Western blot images can be found in Figure S4. (**I**) qRT-PCR analysis of *FBXO32*, *TRIM63*, and *MYOG* mRNA levels in MFLCs of each group. (**J**) Western blot analysis of FBXO32, TRIM63, and MYOG protein levels transfected with an miR-335-5p or NC inhibitor in MFLCs. (**K**) qRT-PCR analysis of *FBXO32*, *TRIM63*, and *MYOG* mRNA levels in MFLCs of each group. *n* = 3–4. The results are expressed as the mean ± SEM. * *p* < 0.05, ** *p* < 0.01, values significantly differ from those of the corresponding control, determined using an unpaired *t*-test. Original Western blot images can be found in Figure S4.

Using the TargetScan and miRanda databases, we predicted the potential target genes of miR-335-5p. Dual-luciferase reporter assays were performed to validate the regulatory effect of miR-335-5p on MAPK11. As shown in Figure 5A, the overexpression of miR-335-5p significantly reduced the activity of the MAPK11-3′UTR-WT reporter gene, whereas the mutated reporter gene showed no significant change in activity. This confirms that MAPK11 is a regulatory target of miR-335-5p, which binds to the 3′UTR of the *MAPK11* gene. To further verify the regulation of MAPK11 by miR-335-5p, we designed and synthesized an miR-335-5p mimic and inhibitor, which were transfected into MFLCs. The results showed that the miR-335-5p mimic exhibited high expression (Figure 5B), whereas the miR-335-5p inhibitor exhibited low expression (Figure 5E). MAPK11, a member of the P38 MAPK family, is known to induce iNOS expression upon activation [22]. When the miR-335-5p mimic was transfected into MFLCs, MAPK11 expression was suppressed, and iNOS expression was similarly reduced (Figure 5C,D). Conversely, transfection of the miR-335-5p inhibitor into MFLCs resulted in the upregulation of MAPK11 expression, with a concurrent increase in iNOS expression (Figure 5F,G). Additionally, transfection of the miR-335-5p mimic into MFLCs led to a significant upregulation of FBXO32 and TRIM63 and a marked decrease in MYOG expression (Figure 5H,I). In contrast, transfection of the miR-335-5p inhibitor into MFLCs resulted in the downregulation of FBXO32 and TRIM63, along with an increase in MYOG expression (Figure 5J,K). These findings indicate that miR-335-5p targets MAPK11/iNOS, contributing to protein degradation in MFLCs.

### 3.6. MSC-Derived EVs Carrying miR-335-5p Induce Protein Degradation in MFLCs via the MAPK11/iNOS Pathway

**Figure 6:**
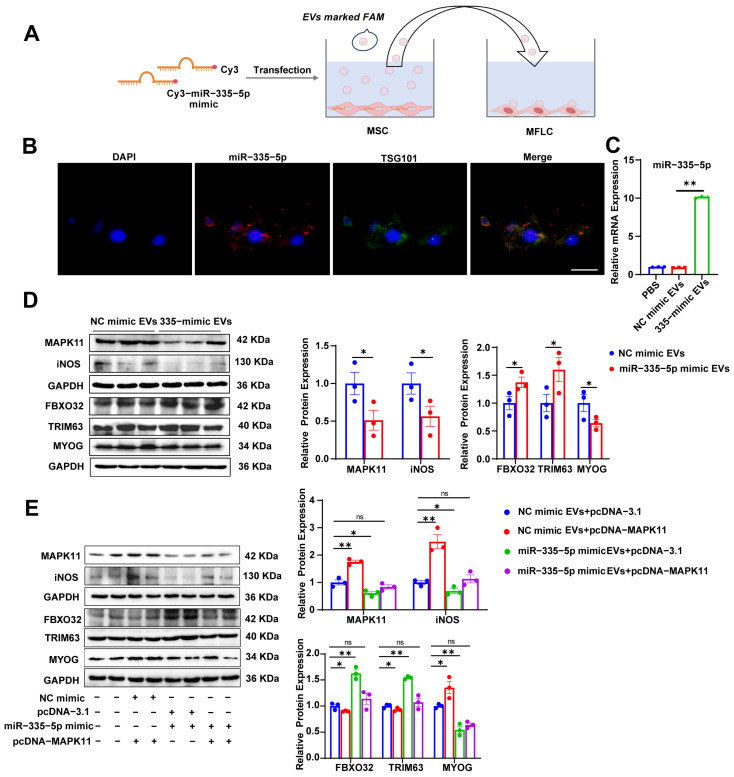
MSC-derived EVs carrying miR-335-5p induce protein degradation in MFLCs via the MAPK11/iNOS pathway: (**A**) A schematic diagram of miR-335-5p or MSC-derived EVs labeled with Cy3 or FAM treated with MFLCs. (**B**) MSCs were transfected with an miR-335-5p NC or miR-335-5p mimic, and MSC-derived EVs were collected representative images of Cy3-labeled miR-335-5p (red) and FAM-labeled TSG101 (green) were obtained after incubation with MFLCs for 24 h. Bar: 50 μm. (**C**) MSCs were transfected with an miR-335-5p NC or miR-335-5p mimic, and MSC-derived EVs were collected; qRT-PCR analysis of miR-335-5p levels after incubation with MFLCs for 24 h. (**D**) Western blot analysis of MAPK11, iNOS, FBXO32, TRIM63, and MYOG protein levels of MSC-derived EVs transfected with miR-335-5p and NC mimics in MFLCs for 24 h. Original Western blot images can be found in Figure S5. (**E**) Western blot analysis of MAPK11, iNOS, FBXO32, TRIM63, and MYOG protein levels in MFLCs transfected with pcDNA-3.1 or pcDNA-MAPK11 and MSC-derived EVs transfected with an miR-335-5p or NC mimic. *n* = 3. The results are expressed as the mean ± SEM. * *p* < 0.05, ** *p* < 0.01, ns, not significant, values significantly differ from those of the corresponding control based on an unpaired *t*-test. Original Western blot images can be found in Figure S5.

To investigate the mechanism by which MSC-derived EVs carrying miR-335-5p regulate MAPK11/iNOS and affect muscle atrophy in MFLCs, MSCs were transfected with an miR-335-5p mimic. EVs were collected from these MSCs and co-cultured with MFLCs (Figure 6A), and the expression of miR-335-5p in MFLCs was analyzed. Confocal laser microscopy detected the expression of miR-335-5p (red fluorescence) and EVs labeled with TSG101 (green fluorescence) in MFLCs. The fluorescence intensity of miR-335-5p in the miR-335-5p mimic EV group was significantly higher than in the NC mimic EV group, whereas TSG101 expression in the NC mimic EV group was markedly higher than in the PBS group (Figure 6B and Figure S1A), the expression level of miR-335-5p detected using qRT-PCR also showed the same trend (Figure 6C). These results indicate the successful internalization of miR-335-5p mimic EVs by MFLCs. The co-culture of EVs derived from miR-335-5p mimic-transfected MSCs with MFLCs increased MAPK11 and iNOS expression in MFLCs (Figure 6D and Figure S1B). Furthermore, EVs from MSCs transfected with the miR-335-5p mimic induced FBXO32 and TRIM63 expression in MFLCs while reducing MYOG expression (Figure 6D and Figure S1C). To validate the targeting association between miR-335-5p and MAPK11 in MSC-derived EVs, MAPK11 overexpression plasmids were designed. EVs from miR-335-5p mimic-transfected MSCs and the MAPK11 overexpression plasmids were co-incubated with MFLCs. The results showed that MAPK11 overexpression plasmids and NC mimic EVs significantly increased MAPK11 and iNOS expression, whereas miR-335-5p mimic EVs reduced their expression. When miR-335-5p mimic EVs and MAPK11 overexpression plasmids were simultaneously added, no differences in MAPK11 or iNOS expression were observed compared to the control group (Figure 6E and Figure S1D). Similarly, adding MAPK11 overexpression plasmids and NC mimic EVs reduced FBXO32 and TRIM63 expression, while increasing MYOG expression. Conversely, the miR-335-5p mimic EVs significantly increased FBXO32 and TRIM63 expression while reducing MYOG expression. When miR-335-5p mimic EVs and MAPK11 overexpression plasmids were added simultaneously, there were no differences in the expression of these markers compared to the control group (Figure 6E and Figure S1E). These findings demonstrate that EVs derived from miR-335-5p mimic-transfected MSCs promote protein degradation in MFLCs by regulating the MAPK11/iNOS pathway.

## 4. Discussion

The prolonged use of GCs leads to muscle atrophy, making it crucial to fully understand the regulatory mechanisms underlying this condition. Our study reveals a novel intercellular communication mechanism between MFLCs and MSCs mediated by iNOS signaling, which regulates GC-induced skeletal muscle atrophy. We demonstrate that Dex-treated MSC-produced EVs enriched with miR-335-5p promote protein degradation in MFLCs through the MAPK11/iNOS pathway, ultimately impairing skeletal muscle development.

Exosomes are membrane-bound nanovesicles secreted by virtually all cell types under both physiological and pathological conditions [23]. Exosomes mediate intercellular communication by delivering regulatory miRNAs that bind to target mRNAs, thereby epigenetically modulating gene expression in recipient cells [24,25]. EVs play a crucial role in signal transduction between muscle cells and other cells, inducing phenotypic changes in target cells [26]. Exosomes derived from skeletal muscle in high-fat diet-fed animals induce myoblast proliferation and regulate the expression of genes associated with the muscle cell cycle and differentiation in vitro [27]. Dex-induced EVs derived from MSCs promote protein degradation in MFLCs and impair skeletal muscle development, confirming the role of myogenic EVs in intercellular communication within the skeletal muscle microenvironment.

iNOS and its catalytic product nitric oxide play crucial roles in various pathological processes in animals, including inflammation, infection, cancer, and myopathy. It has been demonstrated that NOS inhibition substantially diminishes the rapid reaction of MSCs to damage [28]. In the presence of GCs, iNOS expression is suppressed in bone marrow-derived macrophages [29]. Dex significantly suppresses cytokine-induced iNOS expression in rat vascular smooth muscle cells [30]. This study also revealed that Dex-induced EVs derived from MSCs suppressed iNOS expression in MFLCs, confirming that GC-induced myogenic EVs regulate skeletal muscle atrophy via iNOS-mediated protein degradation.

miRNAs are conserved small non-coding RNAs that post-transcriptionally downregulate gene expression through miRNA–mRNA interactions [31,32]. MyomiRs are indispensable for skeletal muscle development [33]. In MFLCs, luciferase reporter assays identified MAPK11 as a target gene of miR-335-5p. MAPK11 (p38β), a subtype of the MAPK family [34], is ubiquitously expressed in mammals and induces iNOS expression upon activation [22]. MAPK11 supports myoblast proliferation and the initiation of myogenesis [35]. Increased MAPK11 expression has been shown to regulate myogenesis via the MAPK signaling pathway activated by estrogen receptor E2 [36]. Through gain- and loss-of-function studies of miR-335-5p, we demonstrated that miR-335-5p targets MAPK11 in MFLCs to regulate iNOS expression, exacerbating muscle atrophy while suppressing myogenesis. These findings provide novel insights into the miRNA-mediated regulation of skeletal muscle development in animals.

Exosomal miRNAs play crucial roles as therapeutic targets and diagnostic biomarkers in various diseases, including cancer progression and metastasis [37,38], neurodegenerative disorders [39], and neurodegenerative disorders [40], and they participate in skeletal muscle myogenesis [41]. The selective packaging of miR-494-3p into cardiomyocyte exosomes may represent a novel intercellular mechanism exacerbating myocardial fibrosis [42]. Pancreatic stellate cell-derived exosomes are enriched with miR-23a-3p, suggesting their potential involvement in type 2 diabetes pathogenesis [43]. Our bioinformatic analysis confirmed significantly higher miR-335-5p expression in Dex-induced MSC-derived EVs than in normal MSC-derived EVs. Functionally, these Dex-primed EVs delivered miR-335-5p to MFLCs, promoting protein degradation and suppressing myogenesis. This suggests that myogenic EV-enriched miR-335-5p mediates GC-induced skeletal muscle atrophy, potentially through ubiquitin–proteasome activation. Similar exosomal miRNA regulation strategies have shown therapeutic potential in other diseases. Exosomes derived from adipose-derived stem cells (ADSCs) deliver miR-378 to enhance osteogenesis and angiogenesis by targeting Sufu to activate the Shh signaling pathway, thereby attenuating GC-induced femoral head necrosis [44]. Mechanically stimulated myoblast-derived exosomes promote bone marrow mesenchymal stem cell (BMSC) proliferation and osteogenic differentiation via the miR-92a-3p/PTEN/AKT axis, demonstrating therapeutic potential against GC-induced osteoporosis in vivo [14]. These studies support the reduction in muscle toxicity of GCs by regulating EV miRNAs (such as modifying EVs to remove miR-335-5p).

Our study demonstrates that GCs induce muscle atrophy through a mechanism involving MSC-derived EVs, which deliver miR-335-5p to MFLCs and activate MAPK11/iNOS-mediated proteolysis. This finding offers new therapeutic perspectives for Duchenne muscular dystrophy (DMD), a progressive disorder marked by muscle atrophy and the loss of muscle cell function [45]. In DMD pathology, the maintenance of muscle protein homeostasis relies on the equilibrium of the ubiquitin–proteasome system. The miR-335-5p-mediated proteolytic pathway may disturb this balance and aggravate muscle protein degradation. Therefore, suppressing miR-335-5p expression in EVs or interfering with its transfer could facilitate muscle cell repair and regeneration. Additionally, this mechanism accounts for the iatrogenic muscle atrophy associated with GC treatment in DMD. GCs potentially enhance miR-335-5p expression in MSC-derived EVs, promoting muscle protein breakdown and leading to proximal limb muscle weakness. Intervention strategies directed at this pathway might provide dual advantages by both enhancing muscle function in DMD patients and preventing muscle atrophy induced by GC treatment. However, this study is limited to the cellular-level validation of the mechanism and lacks in vivo experiments in DMD animal models. Therefore, we cannot confirm the actual efficacy of targeting miR-335-5p in alleviating GC-induced muscle atrophy. These critical issues need to be thoroughly investigated in follow-up studies to facilitate the clinical translation of these findings.

## 5. Conclusions

In summary, our study uncovers a novel mechanism whereby GC-induced MSCs release miR-335-5p-enriched EVs, triggering proteolytic activation in MFLCs. These results position EV-carried miR-335-5p as a promising therapeutic target for mitigating both Duchenne muscular dystrophy and GC-induced muscle atrophy. Future research should focus on developing targeted delivery strategies and validating these findings in vivo to advance clinical applications.

## Figures and Tables

**Table 1:** mRNA and miRNA primer sequences.

miRNA or Gene Name	Primer Sequences (5′ to 3′)
*iNOS*-F	GTTCTCAGCCCAACAATACAAGA
*iNOS*-R	GTGGACGGGTCGATGTCAC
*β-actin*-F	TTGCTGACAGGATGCAGAAG
*β-actin*-R	ACATCTGCTGGAAGGTGGAC
*FBXO32*-F	CAGCTTCGTGAGCGACCTC
*FBXO32*-R	GGCAGTCGAGAAGTCCAGTC
*TRIM63*-F	GTGTGAGGTGCCTACTTGCTC
*TRIM63*-R	GCTCAGTCTTCTGTCCTTGGA
*MYOG*-F	CAGCTTCGTGAGCGACCTC
*MYOG*-R	GGCAGTCGAGAAGTCCAGTC
*MAPK11*-F	GTGTGAGGTGCCTACTTGCTC
*MAPK11*-R	GAGCAGACTGAGCCGTAGG
miR-335-5p	CGCGTCAAGAGCAATAACGAA
miR-144-3p	GCGCGCGTACAGTATAGATGA
miR-148a-3p	GCGCGTCAGTGCACTACAGAA
miR-20a-5p	CGCGCGTAAAGTGCTTATAGTG
miR-17-5p	GCGCAAAGTGCTTACAGTGC
miR-146a-5p	CGCGTGAGAACTGAATTCCA
U6-F	CTCGCTTCGGCAGCACA
U6-R	AACGCTTCACGAATTTGCGT

## Data Availability

The original contributions presented in this study are included in the article/Supplementary Material. Further inquiries can be directed to the corresponding author.

## Supplementary Materials

The following supporting information can be downloaded at: https://www.mdpi.com/article/10.3390/biom15081072/s1, Figure S1: MSC-derived EVs carrying miR-335-5p induce MFLC protein degradation through MAPK11/iNOS; Figure S2: Original western blot images related to Figure 2; Figure S3: Original western blot images related to Figure 3; Figure S4: Original western blot images related to Figure 5; Figure S5: Original western blot and gel images related to Figure 6.
