# Supplementary material for: Glucocorticoid-Induced Muscle Satellite Cell-Derived Extracellular Vesicles Mediate Skeletal Muscle Atrophy via the miR-335-5p/MAPK11/iNOS Pathway
Source: Biomolecules. 2025 Jul 24;15(8):1072. doi: 10.3390/biom15081072 (PMC12383710; doi:10.3390/biom15081072)

Figure S1

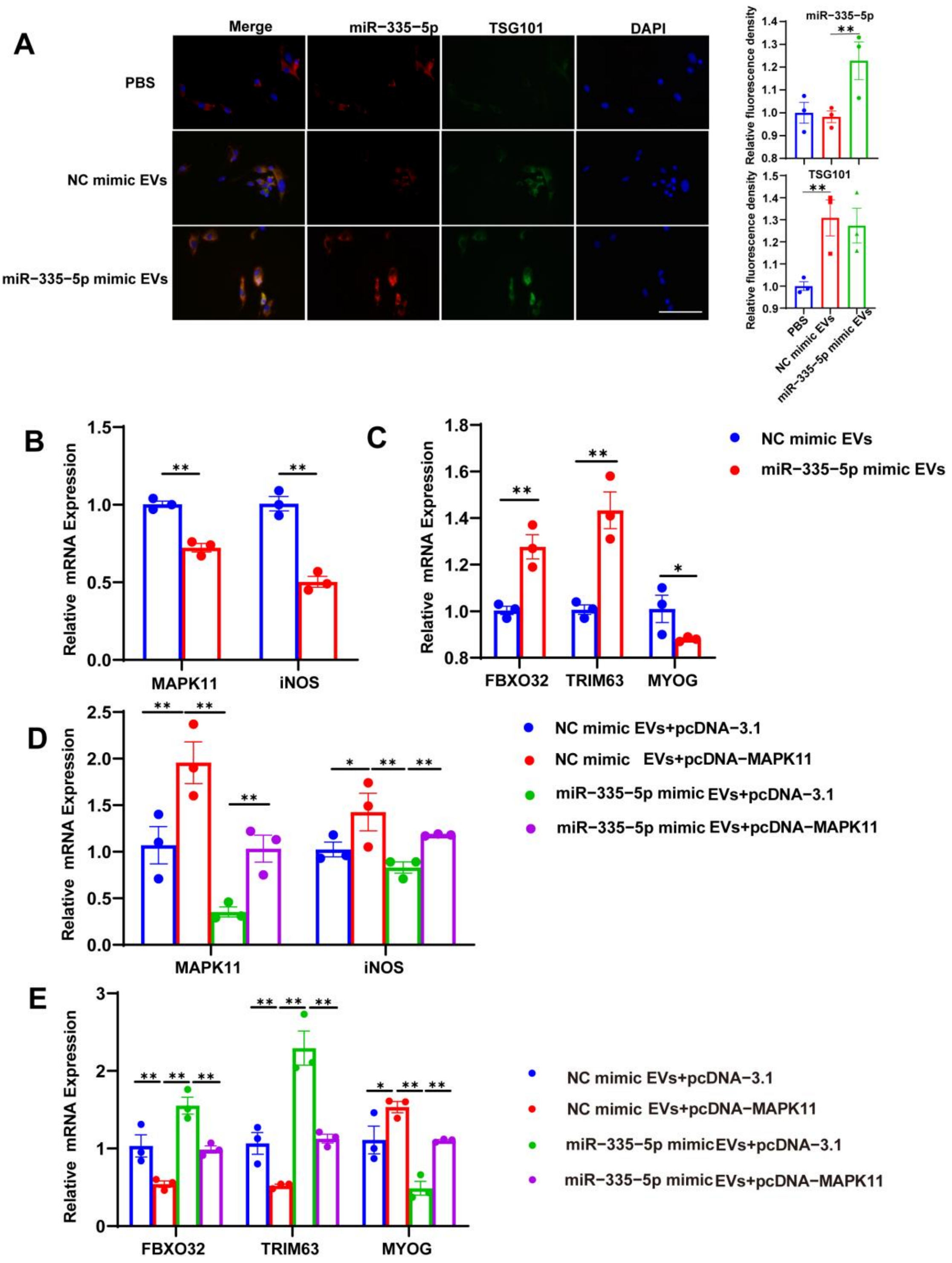

Figure S1. MSC-derived exosomes carrying miR-335-5p induce MFLC protein degradation through MAPK11/iNOS: (A) MSC were transfected with miR-335-5p NC mimic or miR-335-5p mimic, and MSC-derived exosomes were collected respectively, representative images of Cy3-labeled miR-335-5p (red) and FAM-labeled TSG101 (green) were obtained after incubation with MFC for 24 h. Bar: 200 μm. (B) qRT-PCR analysis of *MAPK11* and *iNOS* mRNA levels of MSC-derived exosomes transfected with miR-335-5p mimic and NC mimic in MFC for 24 h. (C) qRT-PCR analysis of *FBXO32*, *TRIM63* and *MYOG* mRNA levels of MSC-derived exosomes transfected with miR-335-5p mimic and NC mimic in MFC for 24 h. (D)

qRT-PCR analysis of *MAPK11* and *iNOS* mRNA levels in MFC transfected with pcDNA-3.1 or pcDNA-MAPK11 and MSC-derived exosomes transfected with miR-335-5p mimic or NC mimic. (E) qRT-PCR analysis of *FBXO32*, *TRIM63* and *MYOG* mRNA levels in MFC transfected with pcDNA-3.1 or pcDNA-MAPK11 and MSC-derived exosomes transfected with miR-335-5p mimic or NC mimic. $n = 3$. The results are expressed as mean ± SEM. $*p < 0.05$, $**p < 0.01$, values signiffcantly different from the corresponding control by unpaired *t*-test.

Figure S2 Original western blot images related to Figure 2

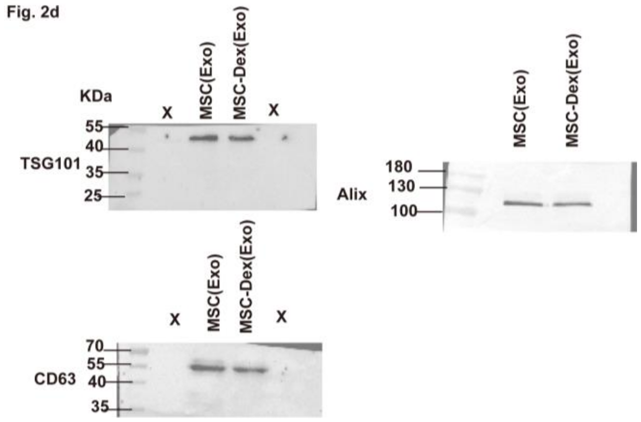

Figure S3 Original western blot images related to Figure 3

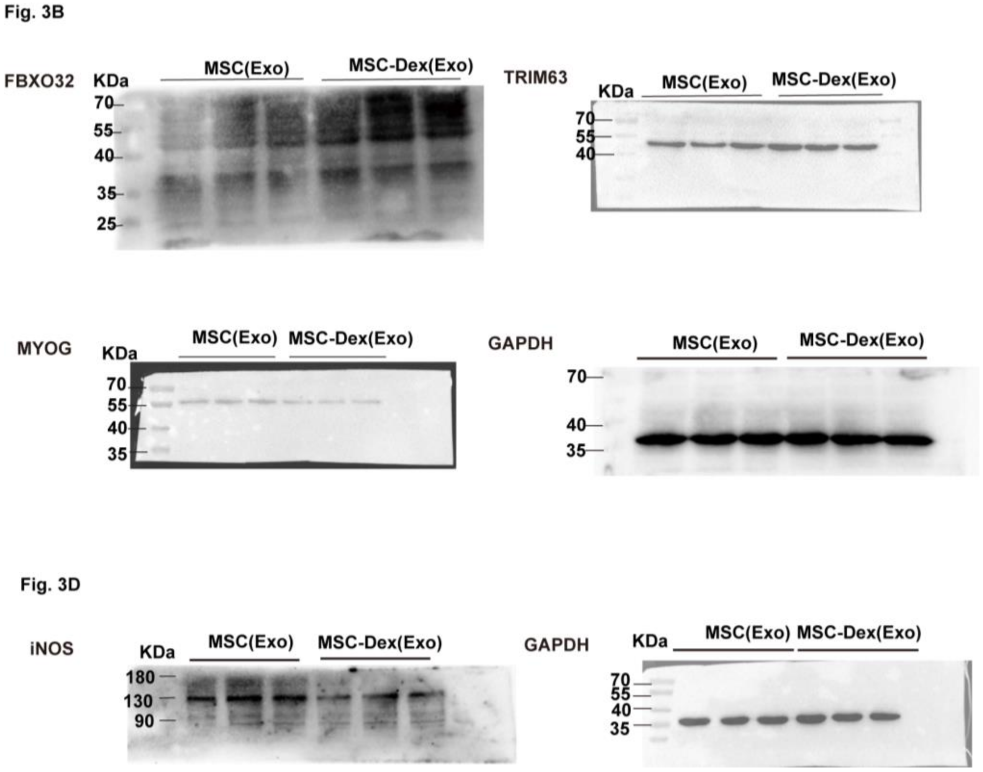

Figure S4 Original western blot images related to Figure 5

Fig. 5C

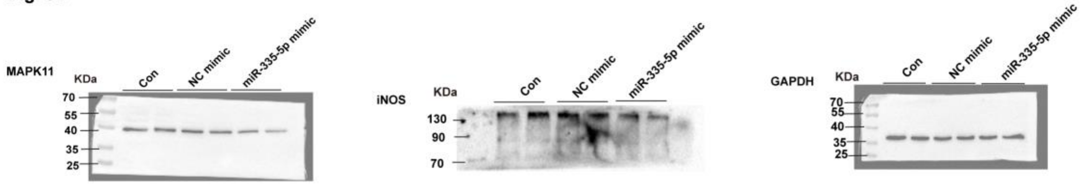

Fig. 5F

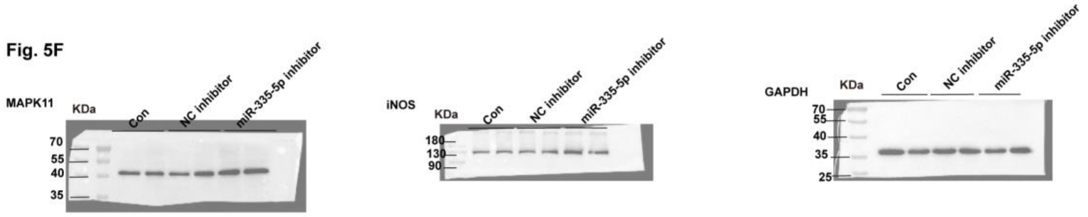

Fig. 5H

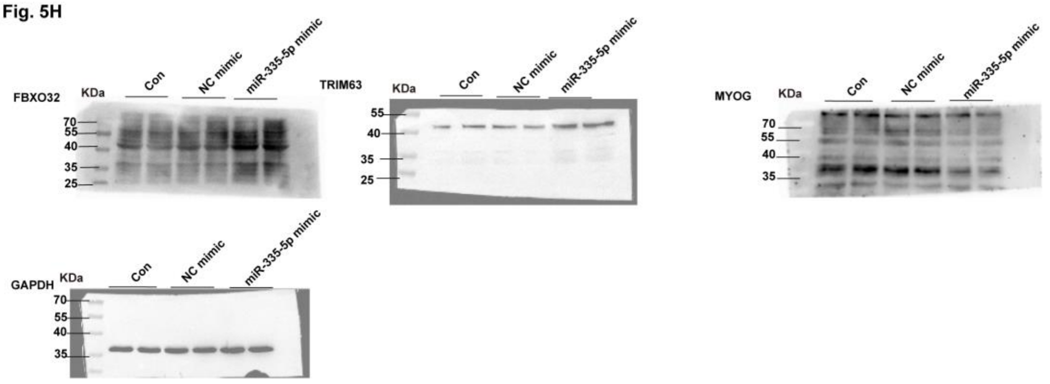

Fig. 5J

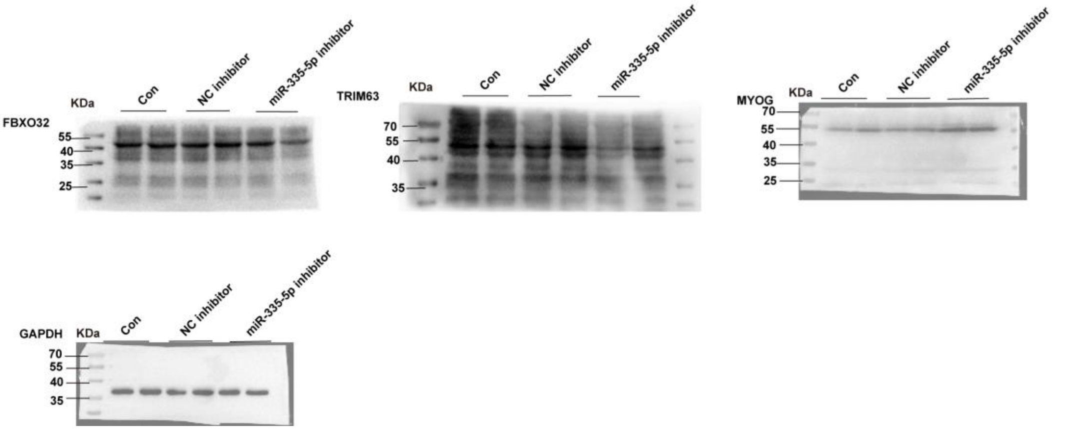

Figure S5 Original western blot images related to Figure 6

Fig. 6D

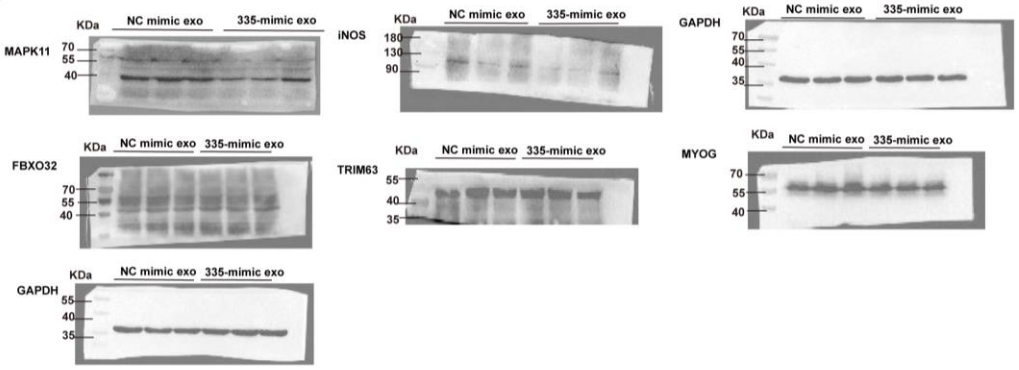

Fig. 6E

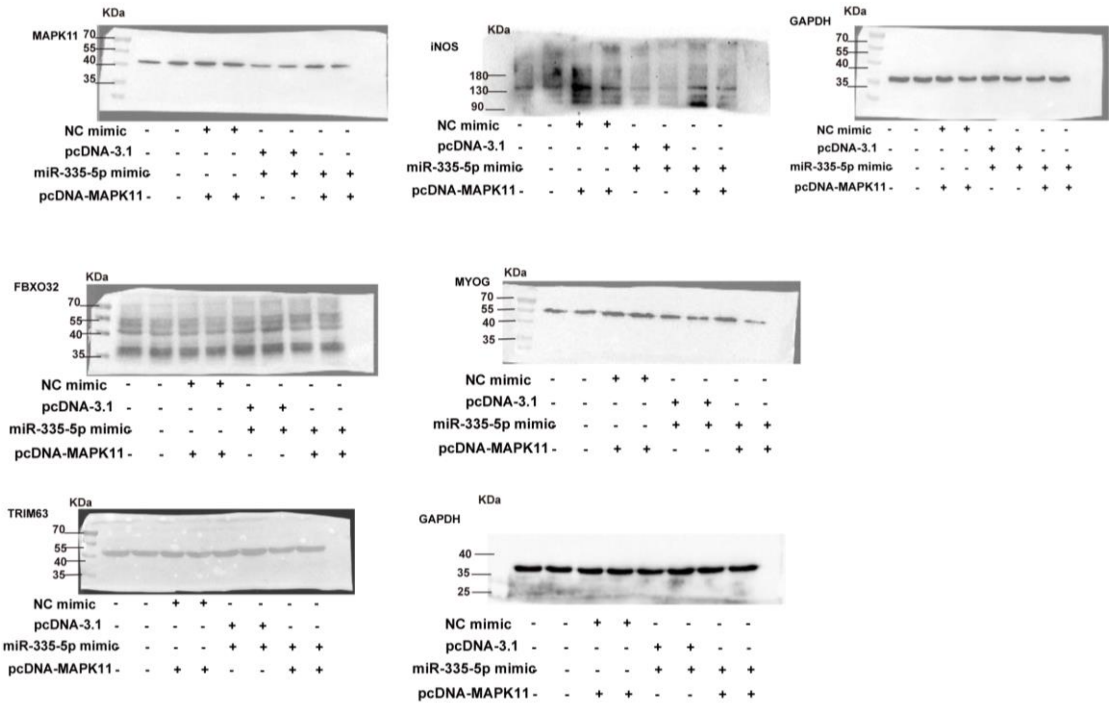

Supplement: Supplementary file 1 [file biomolecules-15-01072-s001.zip › biomolecules-3719068-Supplementary.pdf]
